# Lunacy in Town and Country

**Published:** 1876-04

**Authors:** 


Lunacy in Town and Country.—Dr. John Sibbald, of Edinburgh, at the British Medical Association, exhibited several tabular statements illustrating the statistics of lunacy. The number annually added to the roll of pauper lunatics from among the residents of country parishes is smaller than the number occurring among the residents of towns, being 35 per 100,000 in the one case, and 56 in the other. He showed that this is probably accounted for by a large number of patients requiring only temporary treatment being sent to asylums in towns, who would be allowed to remain at home in the country. He adduced arguments and figures to show that the apparently enormous increase of lunacy in late years was only an apparent increase, and that there is no proof statistically that there is a larger proportion of actual insanity in the population now than there was thirty years since.—*Half-Yearly Compendium,* January, 1876.

